# Effect of grain structure on Charpy impact behavior of copper

**DOI:** 10.1038/srep44783

**Published:** 2017-03-17

**Authors:** Ningning Liang, Yonghao Zhao, Jingtao Wang, Yuntian Zhu

**Affiliations:** 1 Nano Structural Materials Center, School of Materials Science and Engineering, Nanjing University of Science and Technology, Nanjing, 210094, China; 2 School of Materials Science and Engineering, Nanjing University of Science and Technology, Nanjing, 210094, China; 3 Department of Materials Science & Engineering, North Carolina State University, Raleigh, NC 27695-7919, USA

## Abstract

Nanostructured (NS) and ultrafine-grained (UFG) materials have high strength and relatively low ductility. Their toughness has not been comprehensively investigated. Here we report the Charpy impact behavior and the corresponding microstructural evolutions in UFG Cu with equi-axed and elongated grains which were prepared by equal channel angular pressing (ECAP) for 2 and 16 passes at room temperature. It is found that their impact toughness (48 J/cm^2^) is almost comparable to that of coarse grained (CG) Cu: 55 J/cm^2^. The high strain rate during the Charpy impact was found to enhance the strain hardening capability of the UFG Cu due to the suppression of dynamic dislocation recovery. The crack in the CG Cu was blunted by dislocation-slip mediated plastic deformation, while the cracks in the UFG Cu were formed at grain boundaries and triple junctions due to their limited plasticity. Near the crack surfaces the elongated grains in ECAP-2 sample were refined by recrystallization, while equi-axed grains in the ECAP-16 sample grew larger.

Impact/fracture toughness of a material represents its capability to prevent crack propagation. It is usually closely related with plasticity: higher plasticity leads to higher toughness. Strength and toughness do not follow simple reciprocal relationship although there is usually a trade-off between strength and ductility. For structural materials, it is ideal to simultaneously possess both high strength for carrying more load, and high toughness for avoiding catastrophic failure. Charpy impact test is the most common technique for evaluating the impact toughness of materials under high strain rates (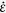
~10^3^ s^−1^)^1^. It has been reported that the impact toughness are affected by the specimen size, notch size, internal defects (inclusion, porosity), microstructure, temperature, etc.^2^,^3^,^4^,^5^. For instance, many materials with body-centered cubic structure exhibit a ductile to brittle transition with decreasing temperature under impact load^6^,^7^,^8^,^9^.

Toughness is an important mechanical property for ultrafine grained (UFG) and nanostructured (NS) materials to be applied in many structural applications^10^. However, literature survey indicates that the toughness of UFG and NS materials has not been well studied and the available results are inconsistent. For instance, in some works, NS and UFG materials are reported to have higher toughness than their coarse grained (CG) counterparts^8^,^9^,^11^,^12^,^13^,^14^,^15^, while other works reported lower toughness^16^. Specifically, grain refinement of Mg alloy from 11 μm to <2 μm increased its impact energy from 10 J to 30 J, which was attributed to higher dynamic strength and plasticity as well as suppression of deformation twinning^12^. Ma *et al*.^13^ attributed the improved impact toughness, from 0.9 to 10 J/cm^2^, of an UFG Al–11%Si alloy to the equal channel angular pressing (ECAP) process, which broke the large aluminum brittle dendrites and inter-dendritic networks. Stolyarov *et al*., found that the impact toughness of NS Ti increased with decreasing temperature, which is opposite to the trend in CG metals and alloys^7^. A steel with low alloy contents and UFG elongated grains as well as nanometer-sized carbides was reported to have an impact energy of 226 J, which is significantly higher than that of its conventional CG counterpart (14 J) at room temperature^6^,^8^. Furthermore, the impact toughness of the NS and UFG materials was found to increase with decreasing temperature^8^. However, reduced impact energy from 9 to 2 J with grain size reduction from 10 μm to 18 nm was observed in cobalt^16^.

These literature results generally show that grain refinement can improve the impact toughness. However, compared with other mechanical properties such as strength and ductility, there has been a lacking of systematic study and understanding on the impact toughness of UFG and NS metals and alloys. In the present paper, we report a systematic investigation on the Charpy impact behavior of bulk UFG Cu with two different grain structures, elongated and equi-axed, produced by ECAP for 2 passes and 16 passes, respectively. For comparison, same investigation was also performed on the CG Cu counterpart.

## Results

### Microstructure

**Figure 1:**
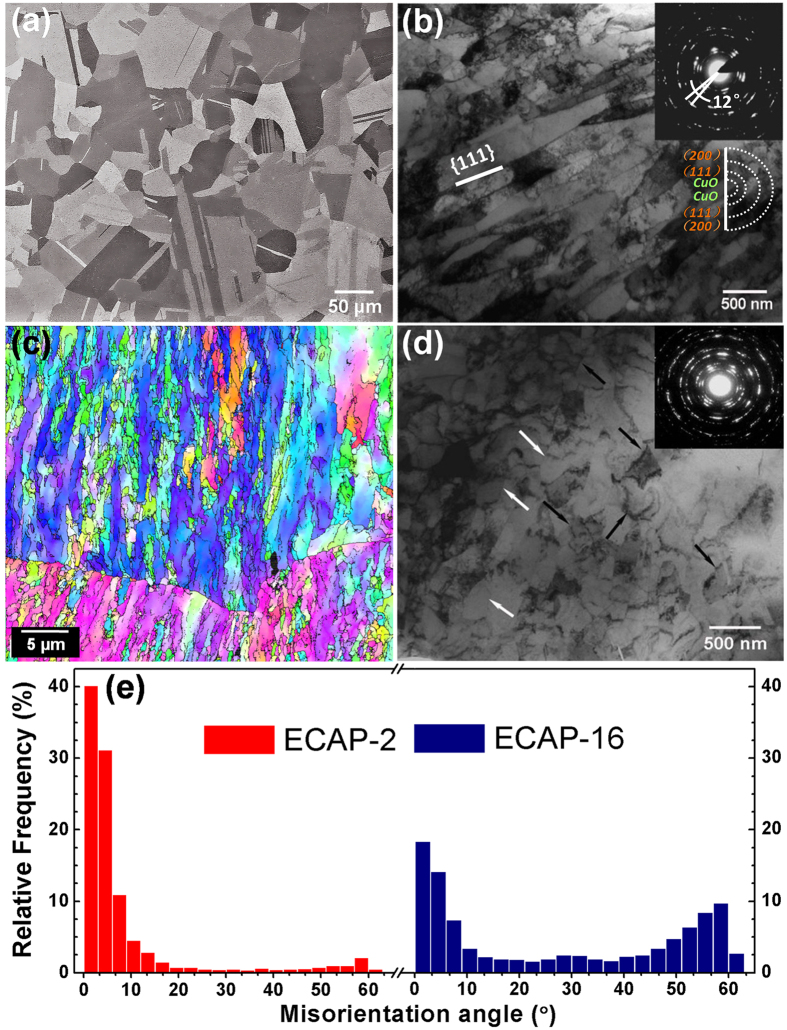
Microstructure characterization of Cu samples. (**a**) Microstructure of initial CG Cu after annealing; (**b**) TEM micrograph of the UFG Cu after ECAP processing for 2 passes. The inset is SAED with a selected area of 5 μm in diameter, (**c**) EBSD map of grain orientation of ECAP-2 Cu. (**d**) TEM micrograph of UFG Cu after ECAP processing for 16 passes. The inset is SAED with a selected area of 5 μm in diameter. (**e**) Distribution of boundary mis-orientation angles measured using EBSD.

The initial CG Cu after annealing was found to have fully recrystallized homogeneous microstructure with equi-axed coarse grains of about 55 μm (Fig. 1a). After ECAP for 2 passes, the coarse grains were divided into parallel elongated grains with boundaries parallel to {111} trace (Fig. 1b). This result suggests that the elongated boundaries are dislocation (111) slip plane with high density of dislocations and low-angle mis-orientation, which was further verified by SAED and EBSD results, as shown in the insets of Fig. 1b and c, respectively. As shown in Fig. 1c, within one coarse grain the color of elongated grains is similar with each other, indicating a small-angle mis-orientation. As shown in Fig. 1b, the spot feature of the SAED pattern from a selected area of 5 micrometer in diameter indicates the crystallographic orientation in this area is similar. While the diffraction spots were spread into an arc shape from which the crystallographic mis-orientation angle was calculated as 12°. The average thickness of the elongated grains is about 230 nm as measured statistically from TEM micrographs, which is consistent with a previous report^17^. After ECAP for 16 passes, homogeneous equi-axed grains with an average grain size of about 270 nm were observed, and they are relatively clean in its interior, possibly resulted from dynamic recrystallization and recovery (pointed by white arrows in Fig. 1d). The ring-like SAED pattern also indicated that the uniform grain distribution with high-angle boundaries^17^,^18^,^19^. In addition, some dislocation cell boundaries are also observed as marked by black arrows. The above microstructural characteristics of ECAP-2 and -16 were further confirmed by grain boundary (GB) mis-orientation angle distribution calculated from EBSD results, as shown in Fig. 1e. The GB mis-orientation angle of ECAP-2 is primarily low angle, while that of ECAP-16 is primarily high angle.

### Mechanical properties

#### Tensile property

**Figure 2:**
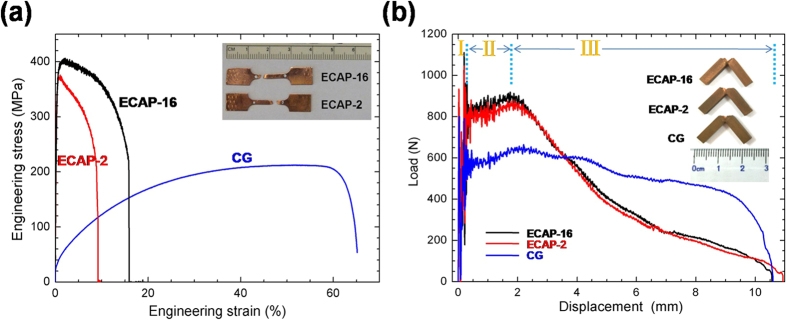
Mechanical properties curves of Cu sample. (**a**) Tensile engineering stress-strain curves of the UFG (ECAP-2 and ECAP-16) and CG Cu at a strain rate of 1 × 10^−3^. (**b**) Load-displacement curves of the UFG (ECAP-2 and ECAP-16) and CG Cu under Charpy notched impact tests at room temperature. The strain rate is 1.3 × 10^3^ s^−1^. Insets show the impacted specimens.

Uni-axial tensile engineering stress-strain curves are shown in Fig. 2a. Compared with the CG Cu, which has yield strength of about 50 MPa and ductility of 50%, the UFG Cu have high tensile strength (370 MPa for ECAP-2 passes, 400 MPa for ECAP-16 passes) and low uniform elongation (1% and 2%) due to the grain refinement and high density of dislocations. Moreover, different from the evident strain hardening of the CG Cu, both ECAP-2 and ECAP-16 Cu necked quickly after yielding because of their low strain hardening capability, as shown in Fig. 2a. The saturated high-density of dislocations and UFG grains of both ECAP-2 and ECAP-16 Cu samples leaves little space for further dislocation accumulation^20^ resulting in geometry softening low uniform elongation as well as low ductility^21^,^22^,^23^.

#### Impact property

Figure 2b shows the impact load-displacement curves of the CG, ECAP-2 and ECAP-16 Cu samples. All impact curves include three stages: I. elastic fluctuation; II. strain hardening stage; III. crack propagation. In stage I, the impact load rose rapidly and fluctuated. This is because when the load just reached the specimen, the impact wave began to spread at the elastic deformation stage. In stage II, the high impact stress activated multiple dislocation sources and led to quick increase in mobile dislocation density. The specimen exhibits a yielding feature at yielding point. With further deformation, the mobile dislocations from different {111} slip systems interacted with each other leading to strain hardening, as shown in Fig. 2b. In stage III, the impact load decreases gradually with crack propagation.

Table 1 lists the impact and tensile testing data of all samples. The CG Cu sample yielded at an impact force of about 565 N, and fractured at 650 N, which corresponds to the onset of crack propagation. However, the UFG Cu samples exhibit much higher yield and fracture forces (790 N and 865 N for ECAP-2; 790 N and 880 N for ECAP-16) caused by the small grain sizes and high density of dislocations. Moreover, in stage III, comparing with the CG Cu sample, which had a stepped and non-continuous load force decrease, the ECAP-2 and ECAP-16 Cu samples have continuous and fast impact load drops, indicating quick crack propagations. The slow and stepped load drop in stage III of the CG Cu indicates there exists large resistance to cracks propagation originated from the crack blunting, which is further resulted from dislocation interactions. The CG Cu has sufficient space for dislocation slip and interactions. In comparison, the UFG Cu has insufficient space for dislocation interaction, and have large amount of GBs for inter-granular crack propagation. This is consistent with literature report that the crack blunting of the UFG materials is not obvious^24^,^25^,^26^, and the stress fields of the arrested dislocations further hamper dislocation emission from cracks in UFG materials. As a result, crack blunting is suppressed, which promoted crack growth.

The absorbed impact energy *A*_*k*_ could be obtained directly from the experiments and the impact toughness *a*_*k*_ could be calculated via normalizing *A*_*k*_ by the cross-section area. Table 1 summarizes *A*_*k*_ and *a*_*k*_ of the CG and UFG Cu. One can see that the *A*_*k*_ and *a*_*k*_ of the UFG Cu (4.4 J, 48 J/cm^2^) are almost as high as those of CG Cu (4.8 J, 55 J/cm^2^), and ECAP-2 and ECAP-16 Cu samples have comparable *A*_*k*_ and *a*_*k*_. The impact energy is proportional to the area of the impact load-displacement curve. Therefore, although the area in stage II of the UFG Cu is larger than that of CG Cu, the overall area of the UFG Cu is still a little smaller than that of the CG Cu because of the smaller area in stage III of the UFG Cu than that of the CG Cu.

Comparing the tensile and impact curves, one can see that there is little strain hardening stage for the UFG Cu subject to the quasi-static tension. However, under impact loading the UFG Cu have strain hardening capability that is comparable with that of the CG Cu. It has been reported that high strain rates can improve the strain hardening capability in UFG/NS materials^27^,^28^,^29^,^30^,^31^,^32^. The enhanced strain hardening capacity at high strain rate can be attributed to the following factors: (i) the high strain rate makes it harder for dislocations to annihilate each other^33^; (ii) high stress activated much more dislocations and enhanced dislocation entanglement; (iii) high strain rate increased the saturation density of dislocations, which made it possible to accumulate more dislocations^34^,^35^.

The experiment also shows that UFG Cu is less resistant to crack propagation (stage III Fig. 2b) than the CG Cu. To investigate the fracture mechanisms behind this observation, we performed SEM and EBSD on the fracture surface and section.

### Fracture crack characteristics

#### Crack of CG Cu

**Figure 3:**
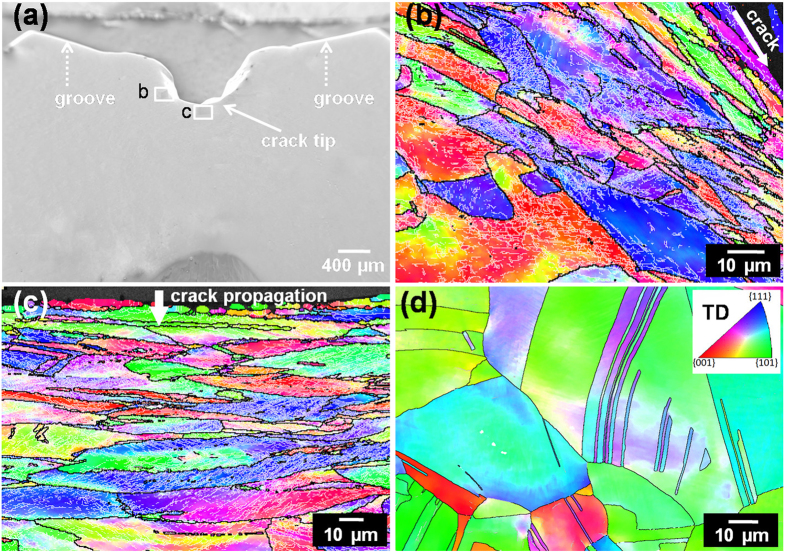
Microstructure characterization of crack section in impacted CG Cu sample. (**a**) SEM image near the crack of a CG Cu sample. (**b**,**c**) OIM images of grains near the crack edge and tip, respectively. (**d**) OIM image of grains far away from the crack. Black lines indicate boundary mis-orientation >15°, and grey lines indicate mis-orientation between 2° and 15°.

Figure 3 shows the SEM and EBSD micrographs of the crack tip of the CG Cu sample after impaction. One can observe a “U” shaped crack, which did not pass through the whole specimen (Fig. 3a). Figure 3b and c illustrate the Orientation imaging microscopy (OIM) images near the impact crack marked by “b” and “c” in Fig. 3a. Severely elongated grains were observed with elongated direction parallel to the crack propagation at the crack edge (Fig. 3b), and perpendicular to the crack propagation direction at the crack tip (Fig. 3c). Moreover, the elongated grains contain a large quantity of low-angle sub-GBs formed from dislocation slip and rearrangement during impact-induced plastic deformation. While equi-axed grains with annealing twins were observed at the position far away from the crack, which are the same as the initial annealed CG Cu (Fig. 3d). These observations further verify the existence of crack blunting via dislocation interaction and grain refinement at the crack tip of the CG Cu, agreeing with the impact load-displacement curve. The grain refinement and dislocation interaction absorbed impact energy and consequently hindered crack propagation.

#### Crack of ECAP-2 Cu

**Figure 4:**
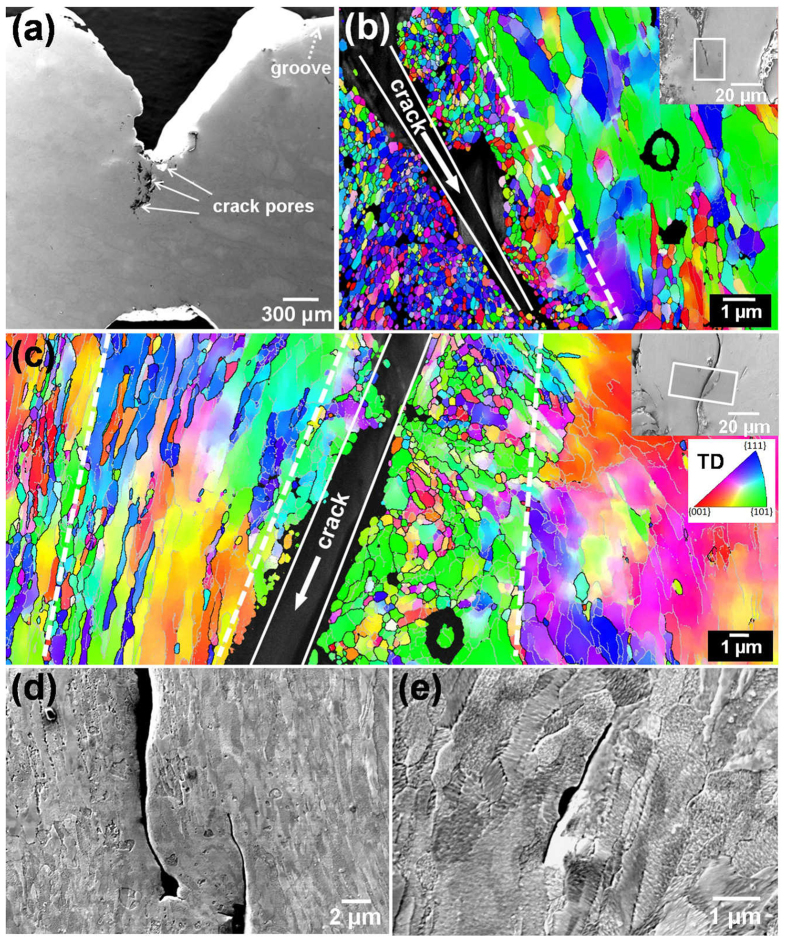
Microstructure characterization of crack section in impacted ECAP-2 Cu sample. (**a**) SEM image of impacted ECAP-2 Cu sample, (**b**) OIM image of grains near crack tip, (**c**) OIM image of grains along the crack edge. Black lines indicate boundary mis-orientation >15°, and grey lines indicate mis-orientation between 2° and 15°. (**d**,**e**) SEM images of small secondary cracks near the main crack of the impacted ECAP-2 Cu sample.

The ECAP-2 Cu sample after impaction has a “V” shaped crack, as shown in Fig. 4a. Moreover, there are some isolate crack pores at the front of the macro V-shaped crack. Further magnified SEM observation found that there exist micro-crack branches at the front of these crack pores. Figure 4b shows the OIM image at the major micro-crack branch (as shown by the inset in Fig. 4b). One can observe the elongated grain structures at the position far away from the crack, which are the same with the as-ECAPed specimen. However, along both side regions near the crack edge, there are zones with a width of several micrometers that is composed of equi-axed grains. These grains have a size range of 0.1 to 0.6 μm which are much smaller than the region far away the crack. This structural evolution came from heat-induced recrystallization due to the quick accumulation of dislocations induced by localized deformation near the crack edge and the local heating up during the high strain rate deformation. Moreover, we found the recrystallized zones are not symmetrical at both sides of the crack. Partial reasons might be as following. During the EBSD sample preparation, the electro-polishing process will corrode more or less the recrystallized area neighboring the crack, which might leave non-symmetrical final observed recrystallized area at both sides of the crack. Moreover, during zigzag crack propagation, the deformation and thermal influence might not equal at both sides of the crack.

Similar results were also observed at the other micro-crack branch, as shown in Fig. 4c. This structural configuration is similar to adiabatic shear band (ASB) in UFG iron^36^ after high strain rate Hopkinson-bar compact, in which grain size was reduced from 0.5 to 0.28 μm in ASB core region. Further SEM observations revealed some isolated short secondary micro-cracks with a length of several micrometers nearby the main micro-crack and parallel to the elongated grain structure (Fig. 4d and e). It was reported^37^ that during cyclic deformation, the fatigue cracks of the ECAP-processed Cu initiated and propagated along the shear plane of the last ECAP pass, which was approximately parallel to the shear bands and boundary of elongated grains^17^. The observed inter-granular crack in Fig. 4d and e suggests that the crack was first nucleated at GBs and propagated along GBs to coalesce together and form a new crack tip. In addition, the initiation of micro voids and crack of the UFG materials mainly occur at GBs and triple junction^38^,^39^,^40^,^41^. These observations also explain the smaller impact toughness of the UFG Cu compared with the CG Cu.

**Figure 5:**
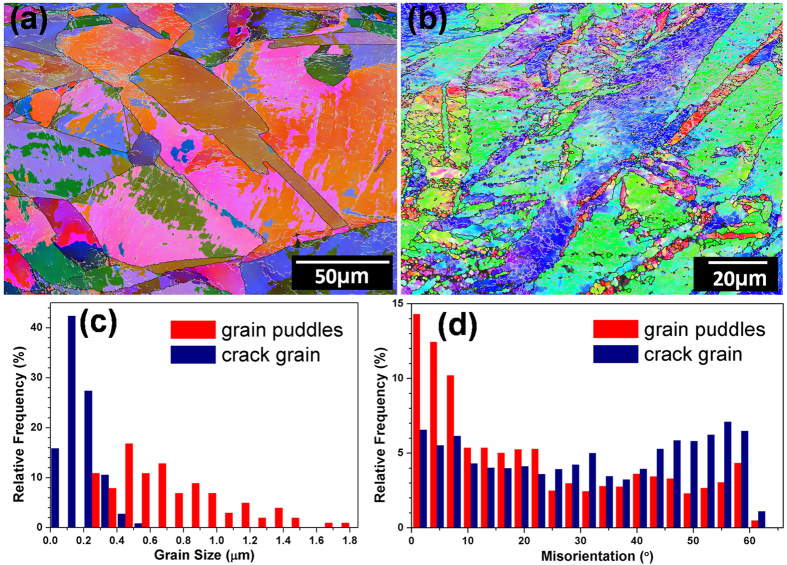
EBSD characterization of ECAP-2 Cu sample with initial grain puddles and impacted crack grains. (**a**,**b**) OIM images of ECAP-2 sample with different magnifications. Frequency distributions of (**c**) grain size and (**d**) GB mis-orientation of as-ECAPed grain puddles and as-impacted near-crack grains.

To further clarify that the UFG grains at the crack zone were formed during impact-induced recrystallization process, but during the ECAP process before impacting, we performed more detailed EBSD analysis, as shown in Fig. 5. The macro-scale homogeneity of the ECAP-2 sample was truly observed by EBSD mapping with large scales, as shown in Fig. 5a and b. Some amount of small grain puddles are surrounded by unclosed GBs and contain dislocations or sub-GBs. Figure 5c and d quantitatively compared the grain size frequency distribution and GB misorientation between the as-ECAPed small grain puddles and the as-impacted near-crack recrystallized grains. Most of the crack grains have the size less than 500 nm, which are much smaller than the grain puddle sizes ranging from 300 nm to 1.8 μm. The average sizes of crack grains and grain puddles are 210 nm and 700 nm, respectively. Moreover, most of the GB mis-orientations of crack grains are high angle, while most GBs of grain puddles are low angle, that is, a typical as-deformed microstructure.

#### Crack of ECAP-16 Cu

**Figure 6:**
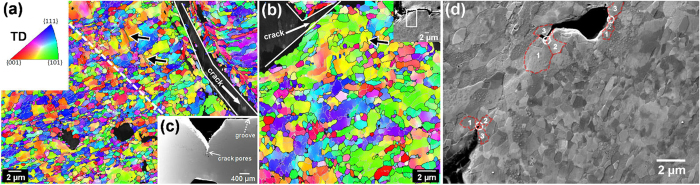
Microstructure characterization of crack section in impacted ECAP-16 Cu sample. (**a**,**b**) OIM images near cracks of the ECAP-16 Cu sample. (**c**) SEM image of impact fractured specimen. Black lines indicate boundary mis-orientation >15°, and grey lines indicate mis-orientation between 2° and 15°. (**d**) SEM image of secondary impact cracks and voids near the main crack of the ECAP-16 Cu sample. Numbered red circles mark the grains around the triple junction.

The impacted ECAP-16 Cu sample also has a “V” shaped crack, as shown in Fig. 6. Figure 6a and b show the EBSD images of grain structures near the crack edges. The sample had equi-axed UFG grains with an average size of 270 nm at the region far away from the crack. However, evident grain growth (grain size ranging from 1 to 4 μm) occurred near the crack edges, as shown in Fig. 6a and b. Moreover, few annealing (growth) twins also appeared as marked by black arrows in Fig. 6a and b. The clean large grains in Fig. 6 also indicate that dislocations in the ECAP-16 Cu sample was annealed away during the impact test, suggesting a significant temperature increase in the crack affected zone. This observation is consistent with what was observed in UFG Cu underwent hat-shaped compression and Taylor tests^42^. Furthermore, the grain structure along the crack is again similar to what is observed in a shear band reported by Mishra *et al*.^42^. Micro-voids and secondary cracks were also observed near the main crack as shown in Fig. 6d. The cracks marked by white circles were inter-granular crack at triple junctions of GBs. Stress concentration at the triple junction promotes void formation and crack propagation. As both the stress level and GB fraction are very high for UFG materials, nano-voids and nano-cracks can be generated at triple junctions due to accumulation of the dislocations resulted from GB sliding^27^,^43^,^44^.

### Temperature rise and thermal stability

Microstructural evolution during the crack propagation is significantly affected by deformation induced heating. For high strain rate deformation, the following formula can be used to calculated temperature rise^18^,^42^,^45^:


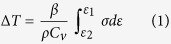


where *β* = 0.9 is the Taylor factor, i.e., assuming 90% of the work of deformation contributes to heating, *ρ* is the sample density and *C*_*v*_ is the heat capacity under constant volume. The area under stress and strain curves from the Charpy impact tests equals absorbed energy per unit volume, i.e., toughness. However, impact toughness as listed in Table 1 is just the absorbed energy divided by the cross sectional area. To be more precise for calculating the temperature rise, the strain energy absorbed per unit volume (*a*) can be described by:


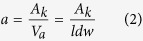


where *A*_*k*_ is impact energy, *V*_*a*_ is impact affected volume, *l* is crack length measured in SEM images of UFG materials in Figs 4a and 5c, *d* is sample thickness (3 mm) and *w* is assumed width of impact affected zone (500 μm as corresponding to the EBSD maps). Then the temperature rise can be calculated as:


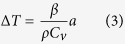


From Mishra *et al*.^42^, *ρ* = 8.97 × 10^3^ kg m^−3^ and *C*_*v*_ = 394 J (kg K)^−1^. The crack length *l* of ECAP-2 Cu sample is about 2.72 mm and impact energy *A*_*k*_ is 4.4 J, so the temperature rise is calculated as Δ*T* = 274 K. As for ECAP-16 Cu, *l* is about 2.64 mm and *A*_*k*_ is 4.4 J, so Δ*T* = 282 K. About 290 °C can be reached for both samples, which is high enough for recrystallization process, as reported by Zhang *et al*.^46^.

It is noted that the temperature rises in ECAP-2 and ECAP-16 samples are close. However, their microstructures near the crack edges after the impact tests are very different. Grain growth was found in ECAP-16 Cu while grain refinement caused by recrystallization was detected in ECAP-2 Cu. These differences of microstructural evolutions during impact were caused by their different initial grain structures. The ECAP-2 Cu has dislocation cell structures. Heating during impaction causes recrystallization at the small-angle GBs or region with high-density of dislocations. In comparison, the ECAP-16 Cu sample has equi-axed recrystallized grains, impact heating directly caused grain growth without nucleation. Liu *et al*.^47^ found the 2D nanometer-scale laminated structure on top layer of bulk Ni with low-angle boundaries is ultra thermo-stable as compared with 3D UFG structure. It is also reported^48^ that the GB mobility increases sharply with increasing misorientation, and the mobility of low-angle boundaries could be 10–500 times lower than that of random high-angle boundaries. Our observation of higher thermal stability of low-angle elongated grains in the ECAP-2 Cu sample is consistent with the above literature reports.

### Fracture surface

**Figure 7:**
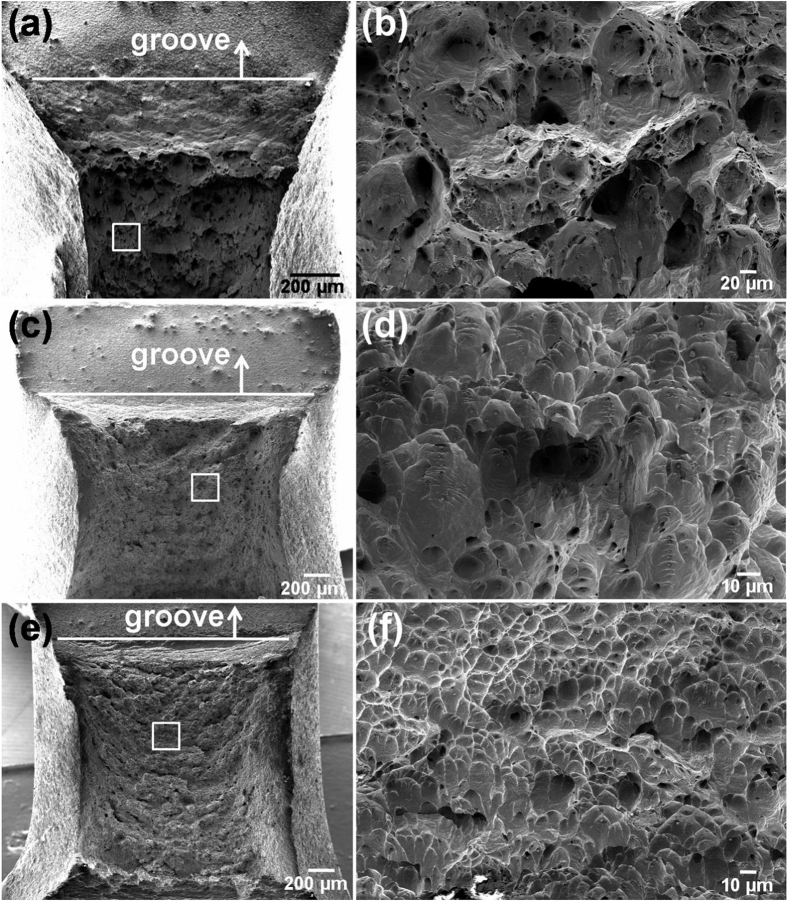
SEM observations of the impact fracture surfaces. CG Cu (**a**,**b**), ECAP-2 Cu (**c**,**d**), and ECAP-16 Cu (**e**,**f**). (**b**,**d**,**f**) are the magnified images of marked area in (**a**,**c**,**e**), respectively.

As shown in Fig. 2b, impact did not separate the Cu samples into two parts indicating a ductile fracture mode. SEM micrographs on the fracture surfaces are shown in Fig. 7. The fracture area of CG Cu sample is apparently smaller than that of ECAP-2 and ECAP-16 Cu samples because sufficient plastic necking occurred in CG Cu sample, while limited necking occurred in ECAPed Cu samples. The area reductions of CG, ECAP-2 and ECAP-16 Cu samples were calculated to be about 62%, 36% and 32%, respectively. The high area reduction of CG Cu sample indicates better plastic deformation ability than that of ECAPed Cu samples, which is consistent with the slow stepped fracture propagation process (Fig. 2b). Meanwhile, larger and deeper uniform dimples (with size measured about 53 μm) were found in the CG Cu sample, as compared with those in the UFG ECAP-2 Cu sample with an average size about 8 μm. The ECAP-16 Cu sample has the smallest and shallowest dimples of all Cu samples with an average size of 4.5 μm (Fig. 7f). These fracture features by impact load is consistent with what were observed under quasi-static tension^49^,^50^.

## Conclusions

In this work, UFG Cu samples were processed by ECAP process for 2 and16 passes, producing elongated grain structure with low-angle GBs and equi-axed grain structure with high-angle GBs, respectively. Both the UFG Cu samples have comparable impact toughness of about 48 J/cm^2^, which is almost comparable with that of the CG Cu samples: 55 J/cm^2^. Compared with the tensile curves under quasi-static strain rate, high strain rate was found to enhance the strain hardening capability of the UFG Cu due to the suppression of dislocation dynamic recovery. EBSD mapping revealed that the CG Cu sample underwent large plastic deformation mediated by dislocation slip in near-crack region, which produced elongated grains and subgrain structure, while the UFG Cu samples formed cracks at the GBs and triple junctions due to limited plasticity and dislocation activity. Along the crack, recrystallized refined grains in the ECAP-2 Cu and large grown grains in the ECAP-16 Cu were found although the temperature rises were close for both samples. The higher thermal stability of the ECAP-2 Cu than ECAP-16 Cu was resulted from both low GB fractions and low-angle GBs.

## Methods

### Sample preparation

As-received pure Cu (99.99%) was annealed at 500 °C for 2 h in a vacuum furnace to produce a CG initial structure with an average grain size of about 55 μm. Square bars with a dimension of 20 × 20 × 80 mm^3^ were machined by electrical discharge. These square bars were processed by ECAP using a die having a channel angle (both exterior and interior) of 90°. Samples were pressed to 2 passes and 16 passes through route *B*_C_ (route B for 2 passes) where the bar was rotated in the same sense by 90° between each pass with 0.4 mm/s velocity at room temperature^51^. For convenience, here we defined the samples after ECAP 2 passes and 16 passes as ECAP-2 and ECAP-16, respectively.

### Mechanical properties testing

Quasi-static uniaxial tensile tests were conducted at room temperature at a strain rate of 1 × 10^−3^ s^−1^. The dog-bone shaped tensile specimens were machined out along the longitudinal direction after ECAP. The gauge dimension is 2 × 1 × 10 mm^3^ and sample surface was polished by 1 μm diamond suspension before tests. The impact toughness was measured by means of Charpy impact tests using a 25 J pendulum instrumented Zwick HIT 50P. The longitudinal direction of the specimens is parallel to the ECAP extrusion direction with dimensions of 3 × 4 × 27 mm^3^. A 60° “V” groove was machined in the middle and the notch depth is 1 mm. To exclude the influence from microstructural heterogeneity of the ECAPed billets, the impact specimens were strictly cut from the central parts of the ECAP billets. The Charpy impact specimen dimensions in this experiment conform to DIN 50 115 standard. At least three successful measurements were used to evaluate the impact toughness for each testing condition. The average impact strain rate is about 1.3 × 10^3^ as calculated the displacement of “V” groove versus impact time.

### Microstructure characterization

Microstructure was characterized using a Philips CM12 transmission electron microscope (TEM) operated at 100 kV. Diffraction patterns were also obtained by selected area electron diffraction (SAED). TEM specimens were cut from the plane parallel to the longitudinal direction of ECAPed bar. These specimen sheets were first grinded step by step using abrasive papers (400 #~1500 #) to <100 μm in thickness and punched to wafers with 3 mm in diameter, then prepared using Ar^+^ ion-milling at 4 kV at temperatures less than 40 °C to get perforation. Moreover, Electron Backscattered Diffraction (EBSD) analysis was performed at the surface areas neighboring fracture section after impact tests. Sample surface was grinded to mirror-like condition and then electro-polished using 2.1 V ac in 85% H_3_PO_4_ + 15% deionized water to remove micro-scratches and relax strained sample surface. EBSD scanning was completed using Zeiss Auriga crossbeam microscope equipped with an Oxford EBSD detector working at 20 KV. Step size chosen for ECAPed samples was 25 nm to obtain accurate resolution for obtaining UFG microstructures. The fracture surfaces of the Charpy impact specimens were also examined by scanning electron microscopy (SEM) integrated in Zeiss Auriga.

## Additional Information

**How to cite this article**: Liang, N. *et al*. Effect of grain structure on Charpy impact behavior of copper. *Sci. Rep.* **7**, 44783; doi: 10.1038/srep44783 (2017).

**Publisher's note:** Springer Nature remains neutral with regard to jurisdictional claims in published maps and institutional affiliations.

## Figures and Tables

**Table 1:** Impact and tensile testing data of Cu samples.

Cu samples	Mechanical properties					
	A_k_ (J)	a_k_ (J/cm^2^)	F_yeild_ (N)	F_max_ (N)	*ε*_u_(%)	*σ*_uts_ (MPa)
CG	4.8 ± 0.1	55 ± 2	65 ± 5	650 ± 5	50	200
ECAP-2	4.4 ± 0.1	48 ± 2	790 ± 5	865 ± 5	1	370
ECAP-16	4.4 ± 0.1	48 ± 2	790 ± 5	880 ± 5	2	400

Absorbed impact energy (A_k_), impact toughness (a_k_), impact yield force (F_yield_) and fracture force (F_fracture_), measured from the Charpy impact tests; uniform tensile elongation (ε_u_), and ultimate tensile strength (uts) measured from the uni-axial tensile tests.
